# Women’s attitudes toward certification logos, labels, and advertisements for organic disposable sanitary pads: results from a multi-city cross-sectional survey

**DOI:** 10.1186/s12905-022-01723-z

**Published:** 2022-06-17

**Authors:** Hayeon Kim, Jinyoung Jung, Yun-Kyoung Song, Taegwon Chang, Sungmin Park, Jiwon Park, Kyungim Kim

**Affiliations:** 1 College of Pharmacy, Korea University, Sejong, Republic of Korea; 2 Institute of Pharmaceutical Science, Korea University, Sejong, Republic of Korea; 3 College of Pharmacy, Daegu Catholic University, Gyeongbuk, Republic of Korea; 4 School of Communication, Daegu Catholic University, Gyeongbuk, Republic of Korea; 5 HnL Law Office, Seoul, Republic of Korea

**Keywords:** Women, Attitude, Safety, Organic disposable sanitary pads, Label, Advertisement, Certification logo

## Abstract

**Background:**

This cross-sectional study evaluated women’s attitudes toward the certification logos, labels, and advertisements for organic disposable sanitary pads (OSPs) and investigated what could be the main reason for them. Additionally, the present study examined whether a relationship could be found between these attitudes and OSPs purchasing behavior.

**Methods:**

This study was conducted using a self-reported online survey of Korean adult women who have purchased OSPs. The study questionnaire had four sections, covering (1) characteristics of OSP purchasing behavior, (2) attitudes toward OSP certification logos, labels, and advertisements, (3) demand on government and companies for proper management, and (4) respondent’s sociodemographic information. The Cronbach’s alpha value of the questionnaire was 0.857.

**Results:**

A total of 500 respondents completed the questionnaire. Overall, high reliability was found for the certification logos (3.73 ± 0.61), labels on the product packaging (3.71 ± 0.63), and advertisements of OSPs (3.41 ± 0.62). Respondents indicated that these had fairly positive effects on their decision-making regarding product reliability, product image, and their own purchasing behavior. The aspects most frequently affected from the informants were safety to human health. All attitudes toward OSP certification logos, labels, and advertisements that were evaluated in this study became more positive in the direction from non-buyers to occasional buyers and to habitual buyers (all *P* < 0.05). The most significant demand from the respondents for OSP companies and the government was to clearly indicate hazardous ingredients on the OSP packaging (42.0%) and to strengthen the sanctions for false advertising (37.8%), respectively.

**Conclusions:**

The results of this study clearly indicate the importance of using certification logos, labels, and advertisements in the OSP market. These results can be utilized by OSP companies to improve the effectiveness of their marketing strategies or by policy makers and certifying bodies to manage the informants properly in the OSP market.

**Supplementary Information:**

The online version contains supplementary material available at 10.1186/s12905-022-01723-z.

## Background

Disposable sanitary pads are considered a necessity for women. Monthly menstruation occurs for about 40 years of the average woman’s life, and most women regularly use disposable sanitary pads during this period. It is estimated that a woman uses approximately 11,400 pads over her lifetime [1]. Because the mucous membrane of a woman’s vagina is in direct contact with the pads, their effect on women’s health has been a constant concern. This concern has rapidly been growing over the past decade as a result of media attention to the potential risks of the chemicals used in disposable sanitary pads for women’s health [2, 3].

As awareness of health issues grows, there are efforts to ensure the safety of disposable sanitary pads for women’s health. In South Korea, the Pharmaceutical Affairs Act of Korea, which addresses medicine and quasi-drugs, has been amended to require that all ingredients used in all such products be indicated on the label of the packaging [4]. New York and California have also enacted similar legislation in the Menstrual Product Right to Know Act of 2019, and the Menstrual Products Right to Know Act of 2020, respectively, to require all ingredients used in pads to be labeled [5, 6]. Women’s interest in and demand for organic disposable sanitary pads (OSPs) has also increased. Analysts forecast that from 2020 to 2024 the global OSP market will grow at a rate of 7% per year for five years [7]. These changes led to an increase in the number of OSP products with certification logos and advertisements highlighting that they are made organically.

Despite the growing use of certification logos, detailed labels on product packaging, and advertisements for OSPs around the world in response to concerns about conventional disposable sanitary pads, few studies have focused on women’s attitudes toward this feature and its impact. Although several studies have evaluated consumers’ perceptions and attitudes toward organic logos or labels for foods [8–12], it is difficult to directly apply these results to OSPs because foods and OSPs have different properties. In this study, we hypothesized that consumer’s attitudes toward the informants—such as OSP certification logos, labels on product packaging, and advertisements—could influence their purchasing behavior. Therefore, we evaluated the women’s attitudes toward the informants and main reasons through an online survey, and investigated whether it was related to purchase decision or consumption frequency.

## Methods

### Study design and participants

This is a cross-sectional self-reported online survey of adult women (details [Checklist for Reporting Results of Internet E-Surveys, CHERRIES] in Additional file 1), with the target population being adult women aged 20 years and over living in Korea who had purchased OSPs. Participants were recruited from a large online panel of approximately 210,000 people managed by a research service company responsible for data collection. To minimize bias, participants went through a two-step process. Initially, panel members were randomly invited to the survey without any information about the subject of the survey. Then, those who wished to participate were directed to the screening page of this survey. During the screening phase, participants were asked four questions to identify adult women who had ever purchased an OSP and those who met the criteria were included in the study sample. Recruitment was stratified by age and state of residence (metropolitan area and province). Informed consent from the participants was obtained online prior to the survey, and each participant was allowed to take part in the survey only once by identifying the client computer’s cookie and IP address. Participants received “points” to a value of about $0.50 from the research service company. Using the Cochran formulas, it was determined that at least 267 survey respondents were required to ensure an appropriately sized sample using item responses on a 5-point scale [13]. The survey was performed from August 26 to September 4, 2020, and all data were encoded to protect the privacy of the survey respondents. The present study is described following the Strengthening the Reporting of Observational studies in Epidemiology (STROBE) statement checklist (Additional file 2).

### Questionnaire

The survey questionnaire was initially developed in Korean based on the relevant literature to identify customer attitudes toward OSP certification logos, labels, and advertisements [14–16]. To ensure content validity, the draft questionnaire was reviewed by experts on the research subject for readability, clarity, and comprehensiveness of each questionnaire item. The intermediate version of the questionnaire was then cognitively tested with 10 adult women and modified following cognitive debriefing and comprehension, interpretation, information summarization, and availability of appropriate responses. Lastly, the developed electronic questionnaire was pilot tested on 20 individuals to ensure the usability and technical functionality. The entire questionnaire for this study is provided as Additional file 3. The Cronbach’s alpha value for the questionnaire was 0.857, indicating excellent reliability.

The questionnaire comprised largely four sections related to purchasing behavior; attitudes toward certification logos, labels, and advertisements; consumer demand for government and companies; and sociodemographic factors. It comprised a total of 37 items; however, because the adaptive questioning was used, not all respondents answered all the items. One item was displayed on each online survey page. Further, all questionnaire items except those required for adaptive questioning were regarded as mandatory. Respondents were unable to change their responses once submitted.

#### Purchasing behavior of OSPs

In this section, respondents were asked “How did you first learn about OSPs?”; “How do you usually buy OSPs?”; “Of each 10 times that you buy disposable sanitary pads, how often do you choose OSPs?”; “When you buy a disposable sanitary pad, what criteria do you use to determine that the product is organic?”; “When you buy an OSP, do you usually check which part of the OSP (e.g., top sheet, absorbent layer, etc.) contains organic ingredients?”; “When you buy an OSP, what do you expect most?” Respondents who answered that they had purchased OSPs one time were considered “non-buyers,” those who bought them from 2 to 6 times were “occasional OSP buyers,” and those who had bought them 7 to 10 times were “habitual OSP buyers.” These values were drawn from previous studies that dealt with targeted and tailored communication strategies regarding different buying-frequency consumer groups [17, 18].

#### Attitudes toward certification logos, labels, and advertisement

In this section, respondents were asked about their attitudes toward OSP certification logos, labels, and advertisements. Questions included “Do you trust certification logos/labels/advertisements of OSP products? (if not, why?),” “Do the certification logos/labels/advertisements of OSP products create a positive image for the product? (if so, in what aspect?),” “Do the certification logos/labels/advertisements of OSP products make you build trust in the product? (if so, in what aspect?),” and “Do the certification logos/labels/advertisements of OSP products affect your decision to purchase the product?” In addition to these, respondents were asked other questions in separate parts: for certification logos, “Do you know the meaning of the certification logos of OSP products?” for labels, “Do you read what the label says?” “Do you understand what the label says?” and “Do you perceive the label’s content to be important?” for advertisements, “Have you ever seen or heard advertisements for OSPs?” “How do you usually see or hear advertisements for OSPs?” and “What interests you the most when you see or hear advertisements for OSP products?” For these questions, a 5-point interval scale was used; “very unlikely” (1), “unlikely” (2), “neutral” (3), “likely” (4), and “very likely” (5). A higher score indicates a more positive attitude, and in this study, 3-point was considered as a cut-off value.

#### Respondents’ demands on government and companies

In this section, the respondents were asked about their demand of the government and companies regarding the appropriate management of certification logos, labels, and advertisements of OSP products.

#### Socio-demographics

In this section, respondents were asked about their age, residence, and educational background.

### Statistical analysis

After the survey results were collected, errors and missing data were reviewed, and only the completed questionnaires were coded into a file for statistical analysis. Categorical variables were presented as values and percentages and compared using Pearson’s chi-square tests. Continuous variables were summarized as mean and standard deviation (SD). Analysis of Variance (ANOVA) was used to compare the differences between the three purchase-frequency groups consisting of continuous variables, and the Bonferroni correction was applied. Two-sided *P*-values less than 0.05 were considered to represent statistical significance. All analyses were performed using SAS 9.4 (SAS Institute, Inc., Cary, NC, USA).

## Results

### Responder characteristics

A total of 500 adult women completed the questionnaire and were included in the analysis. Table 1 presents a summary of the respondents’ characteristics. Respondents by age decade from their 20 s to their 50 s participated at similar rates (around 20%), while those in their 60 s and older were a relatively low proportion of the total (9.6%). More than half of the respondents (58.0%) were living in a metropolitan area, and all of the others were living in other provinces in Republic of Korea. About two-thirds of the respondents (67.4%) had completed university or above. There were insignificant differences in respondents’ characteristics among the three purchase-frequency groups.

**Table 1:** Characteristics of the survey respondents, N (%)

Characteristics	Total (n = 500)	Frequency of purchase			
		Non-buyer (n = 52)	Occasional buyer (n = 286)	Habitual buyer (n = 162)	*P* value
Age (years)					0.340
20–29	99 (19.8)	13 (13.1)	56 (56.6)	30 (30.3)	
30–39	107 (21.4)	14 (11.1)	53 (61.2)	40 (34.7)	
40–49	119 (23.8)	14 (12.4)	67 (68.1)	38 (38.6)	
50–59	127 (25.4)	6 (4.7)	82 (64.6)	39 (30.7)	
≥ 60	48 (9.6)	5 (10.4)	28 (57.2)	15 (32.4)	
Residential area^a^					0.610
Metropolitan area	290 (58.0)	30 (10.3)	161 (55.5)	99 (34.1)	
Provinces	210 (42.0)	22 (10.5)	125 (59.5)	63 (30.0)	
Education level completed					0.945
High school or below	96 (19.2)	11 (11.5)	56 (58.3)	29 (30.2)	
Junior college	63 (12.6)	8 (12.7)	34 (54.0)	21 (33.3)	
University	297 (59.4)	29 (9.8)	171 (57.6)	97 (32.7)	
Graduation school	40 (8.0)	3 (7.5)	23 (57.5)	14 (35.0)	
Other	4 (0.8)	1 (10.4)	2 (57.2)	1 (32.4)	

^a^Residental area was categorized into metropolitan area (Seoul, Incheon, and Gyeonggi) and provinces (Daejeon, Sejong, Chungnam, Chungbuk, Gwangju, Jeonnam, Jeonbuk, Busan, Ulsan, Gyeongnam, Daegu, Gyeongbuk, Gangwon, and Jeju)

### Purchasing behavior on OSP

The average number of OSP purchases of the last 10 times was 5.06 (± 2.95). Approximately 10% of respondents were identified as non-buyers, while 57.2% and 32.4% of respondents were classified as occasional and habitual buyers, respectively. As shown in Table 2, the most common way that to first learned about OSPs was by looking at the label or statements on the packaging (38.7%) or manufacturers’ broadcast or print advertisements (37.8%). Nearly all of the respondents purchased OSPs from an e-commerce market (55.3%) or in-store (43.9%). About half of the respondents said that they judged a product to be organic when they saw a certification logo on the packaging. More than two-thirds of the respondents (70.2%) checked which part(s) (e.g., top sheet, absorbent layer, etc.) of the OSP product was or were made from organic ingredients. When the respondents purchased OSPs, their highest expectation was human safety (77.4%).

**Table 2:** Respondents’ purchasing behaviors on organic disposable sanitary pads

Item	No (%)
Way of first learning about OSPs^a^	
Labels or statements on the product packaging observed in store	194 (38.7)
Manufacturers’ broadcast or print advertisements	189 (37.8)
Recommendations from others (family or other buyers)	84 (16.9)
Celebrity advertisements sponsored by manufacturers	26 (5.2)
Else	7 (1.4)
Main way to purchase OSPs^a^	
E-commerce market	277 (55.3)
In-store shopping	220 (43.9)
Else	3 (0.7)
Criteria used to determine a product as an OSP^a^	
Certification logo on the product packaging	228 (45.6)
Promotional statements described on the product packaging	118 (23.6)
Ingredient information in the label on the product packaging	102 (20.4)
Promotional statement of the product advertisement	51 (10.4)
Other	1 (0.1)
Whether to check which parts of OSPs are made from organic ingredients	
Yes	351 (70.2)
No	149 (29.8)
What to expect from OSPs	
Safety for the human body	387 (77.4)
Excellent absorbency	54 (10.8)
Comfortable to wear	51 (10.2)
Environmental friendliness	8 (1.6)

OSP, organic disposable sanitary pads

^a^Multiple responses available

### Attitudes toward certification logos, labels, and advertisements of OSPs

For the OSP certification logo, the average score for the respondents’ awareness was 3.09 (± 0.73), and there was a significant difference among the three purchase frequency groups (*P* < 0.001). Habitual consumers had the highest awareness (3.28 ± 0.73), followed by occasional buyers (3.07 ± 0.69) and non-buyers (2.62 ± 0.72). All the items asking about the attitude toward the certification logo were rated above 3, which can be interpreted as a positive attitude of consumers toward the OSP certification logo (Table 3). The respondents felt reliability in OSP certification logos (3.73 ± 0.61) and thought that the certification logos highly contributed to a positive image of the product (4.05 ± 0.64). For this positive image, the most affected aspect was safety for the human body (88.4%), followed by environmental friendliness (6.1%), comfort in wearing (3.7%), and excellent absorbency (1.8%). In addition, the OSP certification logos had a positive effect on the sense of the reliability of the product itself (3.91 ± 0.61), especially in terms of safety for the human body (86.7%), and it also had a strong influence on the product purchase decision (3.93 ± 0.67). Attitudes toward the certification logos of the OSPs became more positive moving from non-buyers to occasional buyers and to habitual buyers (all *P* < 0.001).

**Table 3:** Respondents’ attitudes toward certification logos, labels, and advertisements of organic disposable sanitary pads, mean (SD)

Item	Total (n = 500)	Frequency of purchase			
		Non-buyers (n = 52)	Occasional buyers (n = 286)	Habitual buyers (n = 162)	*P* value
Certification logos					
Trust	3.73 (0.61)	3.19 (0.74)^a^	3.73 (0.56)^b^	3.89 (0.54)^c^	< .001
Positive image	4.05 (0.64)	3.65 (0.86)^a^	4.03 (0.58)^b^	4.22 (0.59)^c^	< .001
Impact on product trust	3.91 (0.61)	3.50 (0.87)^a^	3.90 (0.56)^b^	4.06 (0.54)^c^	< .001
Impact on purchase	3.93 (0.67)	3.50 (0.75)^a^	3.88 (0.64)^b^	4.17 (0.59)^c^	< .001
Labels					
Trust	3.71 (0.63)	3.27 (0.69)^a^	3.71 (0.58)	3.84 (0.64)	< .001
Impact on product trust	3.74 (0.66)	3.35 (0.81)^a^	3.71 (0.58)^b^	3.91 (0.66)^c^	< .001
Impact on purchase	3.56 (0.81)	2.94 (0.94)^a^	3.57 (0.73)	3.73 (0.82)	< .001
Advertisements^d^					
Trust	3.41 (0.62)	2.97 (074)^a^	3.43 (0.59)	3.48 (0.59)	< .001
Positive image	3.64 (0.63)	3.41 (0.79)	3.64 (0.62)	3.72 (0.60)	0.026
Impact on product trust	3.54 (0.63)	3.21 (0.80)^a^	3.53 (0.59)	3.62 (0.62)	0.001
Impact on purchase	3.58 (0.72)	3.26 (0.94)^a^	3.58 (0.63)	3.66 (0.76)	0.007

^a–c^Scores within a row with different superscripts indicate significantly different means using Bonferroni post hoc

^d^The total number of respondents is 435, including those who had ever seen or heard an OSP advertisement

Regarding the label on the OSP product packaging, a positive attitude similar to that of the certification logos was found (Table 3). The respondents indicated that they had high trust in the labels on the OSP product packaging (3.71 ± 0.63) and that the labels made the product itself trustworthy (3.74 ± 0.66). The most affected aspect regarding product reliability was its safety for the human body (89.9%), followed by its environmental friendliness (6.1%), comfort in wearing (3.7%), and excellent absorbency (1.8%). The labels on the OSP product packaging also had an influence on the product purchase decision (3.56 ± 0.81). The three purchase frequency groups also differed in their attitudes toward labels on OSP product packaging, which were more favorable in proportion to frequency (all *P* < 0.001). Regarding the labels, the respondents’ attitudes toward the detailed contents of the labels were also identified (Table 4). The respondents indicated that the expiration date, ingredients, and usage precautions were the most important among the label contents on the product packaging, followed by masses of manufacturer information and storage methods (3.84 ± 0.82, 3.79 ± 0.87, 3.61 ± 0.92, 3.42 ± 0.91, and 3.36 ± 0.86, respectively). The respondents were also likely to read the expiration dates (3.36 ± 1.03), ingredients (3.25 ± 1.00), usage precautions (3.20 ± 1.02), manufacturer information (3.17 ± 1.00), and storage methods (2.93 ± 0.95). The respondents' level of understanding of label contents was highest for the expiration date (3.77 ± 0.83), followed by usage precautions (3.59 ± 0.88), manufacturer information (3.49 ± 0.89), storage methods (3.43 ± 0.89), and ingredients (3.28 ± 0.90). The attitudes toward the details of the label showed significant differences between the non-buyers, occasional buyers, and habitual buyers, with the exception of the importance assigned to the storage methods and usage precautions, and the degree of reading of the usage precautions (Table 4).

**Table 4:** Respondents’ attitudes toward the details of organic disposable sanitary pads’ labeling, mean (SD)

Item	Total (n = 500)	Frequency of purchase			
		Non-buyers (n = 52)	Occasional buyers (n = 286)	Habitual buyers (n = 162)	*P* value
Importance					
Overall	3.60 (0.66)	3.30 (0.78)^a^	3.60 (0.62)	3.70 (0.65)	0.001
Expiration date	3.84 (0.82)	3.46 (0.90)^a^	3.84 (0.78)	3.96 (0.83)	0.001
Manufacturer	3.42 (0.91)	3.02 (0.98)^a^	3.39 (0.90)	3.59 (0.87)	< .001
Ingredients	3.79 (0.87)	3.52 (0.98)	3.74 (0.87)	3.95 (0.82)^c^	0.004
Storage	3.36 (0.86)	3.13 (0.93)	3.38 (0.84)	3.39 (0.88)	0.146
Usage precautions	3.61 (0.92)	3.38 (1.07)	3.64 (0.89)	3.62 (0.93)	0.179
Reading					
Overall	3.18 (0.77)	2.79 (0.84)^a^	3.16 (0.70)^b^	3.35 (0.64)^c^	< .001
Expiration date	3.36 (1.03)	2.98 (1.15)	3.30 (0.95)	3.59 (1.09)^c^	< .001
Manufacturer	3.17 (1.00)	2.60 (1.00)^a^	3.15 (0.99)^b^	3.40 (0.97)^c^	< .001
Ingredients	3.25 (1.00)	2.75 (1.12)^a^	3.21 (0.96)^b^	3.48 (1.00)^c^	< .001
Storage	2.93 (0.95)	2.65 (1.06)	2.91 (0.90)	3.06 (0.99)	0.026
Usage precautions	3.20 (1.02)	2.96 (1.00)	3.22 (0.98)	3.24 (1.10)	0.206
Understanding					
Overall	3.51 (0.65)	3.15 (0.72)^a^	3.50 (0.59)^b^	3.67 (0.69)^c^	< .001
Expiration date	3.77 (0.83)	3.52 (0.96)	3.76 (0.78)	3.88 (0.87)	0.021
Manufacturer	3.49 (0.89)	3.06 (0.92)^a^	3.47 (0.87)^b^	3.68 (0.88)^c^	< .001
Ingredients	3.28 (0.90)	2.92 (1.05)^a^	3.26 (0.82)	3.45 (0.95)	0.001
Storage	3.43 (0.89)	3.04 (1.00)^a^	3.42 (0.84)	3.57 (0.90)	0.001
Usage precautions	3.59 (0.88)	3.21 (0.94)^a^	3.56 (0.83)	3.75 (0.90)	< .001

^a–c^Scores within a row with different superscripts indicate significantly different means using Bonferroni post hoc

Only those respondents who had ever seen or heard an OSP advertisement (n = 435, 87%) were included in the advertisement analysis. About 90% of the respondents had encountered OSP advertisements, either on the air or online (58.6% and 29.2%, respectively). The respondents had a positive attitude toward the reliability of OSP advertisements (3.41 ± 0.62), and the effects of advertisements on product image (3.64 ± 0.63), product reliability (3.54 ± 0.63), and purchase decision (3.58 ± 0.72) (Table 3). The OSP advertisements contributed the most to the formation of a positive image of the product in terms of its safety for the human body (80.8%) the most, followed by comfort in wearing (9.0%), excellent absorbency (5.2%), and environmental friendliness (4.0%). For the impacts on product reliability, safety for the human body was ranked highest (80.9%), followed by comfort in wearing (8.4%), environmental friendliness (5.3%), and excellent absorbency (4.3%). An increase in OSP purchase frequency was also associated with more favorable attitudes toward OSP advertisements (all *P* < 0.05).

### Demands on governments and OSP companies for proper management

For the proper management of certification logos, labels, and advertisements in the OSP market, the respondents’ foremost demand for OSP companies was to make OSP packaging clear and to allow them to easily identify statements regarding hazardous ingredients (42.0%). The second-ranked demand was to clearly indicate the specific parts (e.g., top sheet, absorbent layers, etc.) of OSPs made from organic ingredients (33.0%) in their advertisements or on the packaging. This was followed by providing detailed information on hazardous ingredients and proof of certification logo acquisition on the product website (15.8% and 9.2%, respectively). For governments, the respondents strongly requested greater sanctions for unfair advertising (37.8%) and the establishment of regulations of the use of certification logos (32.7%). Other demands included consumer education for more intelligent consumption (15.2%) and corporate education to prevent unfair advertising (14.4%).

## Discussion

Women’s demands for OSPs are growing with the expectations for their safety for body. However, it is hard for consumers to verify whether a given product was produced according to the promised characteristics [9, 19] because organic products are representative credence goods. In organic markets, consumers generally rely on information provided by producers, sellers, or independent third parties [10, 20]. Because OSPs, as credence goods, also have properties of asymmetric information, information signaling such as through certification logos and detailed labels on product packaging, as well as advertisements in OSP markets, are important tools that a woman can use to evaluate product quality by converting credence characteristics into search attributes. Therefore, a clear understanding of women’s attitudes toward these informants and their impact on purchasing is important. In this study, women showed a fairly high level of trust in the informants, which were evaluated to have had a positive effect on the product’s reliability, image, and purchase decision. Women have favorable attitudes toward the informants in terms of safety for their health. In addition, it was found that positive attitudes regarding the informants have a relationship with actual OSP purchasing behavior.

This study showed that the sociodemographic characteristics, including age, education background, and residential area, were not significantly different among the three purchase frequency groups. These results are similar to those of previous studies on organic foods [21, 22]. Because the respondents answered that they most expected safety for human health when purchasing OSPs, this result suggests that sociodemographic factors, as in the case of food, may not have a greater impact on consumer purchasing behavior than health concerns. Although respondent incomes were not collected in this study, it is unlikely to have a significant impact on the results, as most previous studies have found that income is not a significant variable in explaining differences in purchasing behavior between buyers and non-buyers of organic products [23].

The respondents in this study showed generally positive attitudes toward the OSP certification logos, labels on the product packaging, and advertisements. The results indicate that women trust those informants, and the informants had positive effects on women’s decisions about the product’s reliability and image, and even on their purchasing behavior. In the credence market, third-party certification or a provision of information via labels or advertisements could be an instrument to gain consumer trust [24]. By this means, customers can be informed of the details of product characteristics through the label on the packaging [25, 26], and has been recognized that the more healthful information that consumers find on the label, the more favorable they become and the more positive their buying decisions become [14, 20, 27]. Certification logos are used to signal consumers at the point of sale that a product is certified. Consumers believe that a certification logo is a proof that the product has satisfied the specific requirements of the given certification, which is controlled by standard regulations, upheld by independent private organizations or governments [9]. Thus, consumers tend to trust certified logos more than statements written by the source company on the product [10]. The effects of the certification logos have been well documented in previous studies on organic food. Toschi et al. reported the presence of the labeling effect; in their experiment, a conventional yogurt with the same odor, taste, and textures as an organic yogurt is evaluated significantly higher when labeled as organic than when it is unlabeled, and vice-versa [28].

In this study, the respondents indicated that the most expected aspect when purchasing an OSP and the aspect that the informants contribute the most to the belief in and impression of the product is safety for human health. However, it is interesting that OSP certification logos, labels, and the word “organic,” used in advertisements, do not guarantee that the product is healthier or that it is free from hazardous ingredients [29–31]. It has not been proven that OSPs are healthier than conventional disposable sanitary pads. This implies that consumers’ beliefs regarding certification logos, labels, and advertisements that emphasize the organic nature of the product outweigh the ambiguity as to whether OSPs are safe. Similarly, one previous study on organic foods reported that the term “organic” carries positive connotations for food, so it can be assumed to feature a heuristic cue or an indicator of perception [32].

Furthermore, this study found that the degree of women’s positive attitude was closely linked to their purchasing behavior. The attitude toward the OSP informants has become more positive going from non-buyers to occasional buyers to habitual buyers. These results suggest that in the OSP market, not only whether women have a positive attitude toward an informant but also the degree of positivity is important. This finding is in line with several previous studies that have found that negative attitudes attributed to uncertainty and lack of trust in organic food logos can act as barriers to purchasing organic food [33–36].

There are some limitations in our study. First, the respondent group was slightly biased toward better educated respondents (about 80% have a degree above college education). This may be attributed to the use of an electronic survey method. Second, this study only focused on OSPs because we observed that, as with previous studies, the most used menstrual hygiene products were disposable menstrual pads, due to their convenience and easiness of discarding [1, 37, 38]. Therefore, women’s attitudes on other kinds of alternatives such as tampons, menstrual cups, cloth menstrual pads should be examined in future studies. Last, caution is required before applying these findings to other countries, as attitudes toward OSPs and their certification logos, labels, and advertisements can vary by social and cultural characteristics.

Despite these limitations, the findings of this study are valuable because, to the best of our knowledge, there has been no previous study that has evaluated women’s attitudes toward OSP certified logos, labels, or advertisements in combination with purchasing behavior. Therefore, although this study was conducted in Korea, it can be used as basic data to understand women’s attitudes toward OSP informants in other countries where the demand for OSP is also increasing.

## Conclusion

This study indicates that certified logos, labels, and advertisements are important tools for ensuring women maintain a positive attitude toward OSP products and key drivers for OSP consumption. The results of this study can contribute to improvements to the effectiveness of the marketing strategies of OSP companies, and it can also provide insight for policy makers or certifying bodies on the importance of the informants and their management in the OSP market.

## Supplementary Information

**Additional file 1. **Checklist for reporting internet E-Survey results. A file entitled “Checklist for Reporting Results of Internet E-Surveys (CHERRIES)".

**Additional file 2. **Checklist for reporting cross-sectional studies. A file named “STROBE statement checklist”.

**Additional file 3. **Contains all the questions of the survey asked for the study participants. A file named "Survey questionnaire"

## Data Availability

All data generated or analyzed during this study are included in this published article and supplementary information files.

## Abbreviation

OSP: Organic disposable sanitary pad
