# Supplementary material for: Women’s attitudes toward certification logos, labels, and advertisements for organic disposable sanitary pads: results from a multi-city cross-sectional survey
Source: BMC Womens Health. 2022 Jun 17;22:237. doi: 10.1186/s12905-022-01723-z (PMC9206355; doi:10.1186/s12905-022-01723-z)
Supplement: Supplementary file 1 — Additional file 1. Checklist for reporting internet E-Survey results. A file entitled “Checklist for Reporting Results of Internet E-Surveys (CHERRIES)". [file 12905_2022_1723_MOESM1_ESM.pdf]

Supplement 1. Checklist for Reporting Results of Internet E-Surveys (CHERRIES)*

Item Category	***Checklist Item***	***Page Number***	***Description***
**Design**	Describe survey design	4	This was an online cross-sectional study. The target population was adult women aged 20 years and over living in Korea who had purchased OSPs. Participants were recruited from a large online panel of about 210,000 people managed by a research service company responsible for data collection.
**IRB (Institutional Review Board) approval and informed consent process**	IRB approval	19	Ethics approval was obtained from the institutional review board of Korea University (IRB No. KUIRB-2020-0208-01).
	Informed consent	4	All participants, as members of the web-based panel, had already provided informed consent to participate in online surveys. Informed consent for this survey was obtained from all those agreeing to participate the survey. On the first page, the study objectives, expected time to complete (approximately 25 minutes), and participant anonymization were explained. The participants were asked to provide informed consent before proceeding to the questions.
	Data protection	19	All data were encoded to protect the privacy of the survey respondents, and the fully de-identified dataset is kept on password protected computers.
**Development and pre-testing**	Development and testing	5	The survey questionnaire was initially developed in Korean based on the relevant literature to identify customer attitudes toward OSP certification logos, labels, and advertisements. To ensure content validity, the draft questionnaire was reviewed by experts on the research subject for readability, clarity, and comprehensiveness of all the questionnaire items. The intermediate version of the questionnaire was then cognitively tested with 10 adult women, and modified following cognitive debriefing and comprehension, interpretation, information summarization, and availability of appropriate responses. Lastly, the developed electronic questionnaire was pilot tested on 20 individuals to ensure the usability and technical functionality.
**Recruitment process and description of the sample having access to the questionnaire**	Open survey versus closed survey	4	This was an open survey that permitted participants to respond if they had the link to the questionnaire.
	Contact mode	4	To minimize bias, participants went through a two-step process. Initially, panel members were randomly invited to the survey without any information on the subject of the survey. Then, those who wish to participate were directed to the screening page of this survey. During the screening phase, participants were asked four questions to identify adult women who had ever purchased an OSP, and those who met the criteria were included in the study sample. Recruitment was stratified by age and state of residence (metropolitan area and province).

	Advertising the survey	4	The survey was not advertised. (Same as above)
**Survey administration**	Web/E-mail	4	In this study, a web-based survey was conducted. Respondents channeled to the online survey site through the survey company's own participant interface ('DATAIN'). Responses were collected via the online survey platform and stored on a secure local server. Responses were multiple choice, numbers, and text.
	Context	4	This survey was not posted publicly. The panel provider is a research services company that maintains a pool of approximately 210,000 potential respondents for research projects.
	Mandatory/voluntary	4	This survey was voluntary. Those who wish to participate in this survey were directed to the screening page. During the screening phase, participants were screened for meeting the inclusion criteria.
	Incentives	4	Participants were incentivized with "points" that could be redeemed for cash from a research service company. The point value was estimated at $0.50.
	Time/Date	5	This study was conducted from August 26 to September 4, 2020,
	Randomization of items or questionnaires	N/A	No randomization of items was used.
	Adaptive questioning	5, Additional file 3	Adaptive questioning was used. Some items were displayed conditionally based on responses to relevant previous questions. For example, only those who did not trust the OSP certification logos were given a follow-up question asking why.
	Number of Items	5	A total of 37 items consisted of the entire questionnaire, although because the adaptive questioning was used, not all respondents answered all the items.
	Number of screens (pages)	5	One item was displayed on each online survey page. And the whole survey, including welcome page, screening page, etc., was distributed over about 50 pages.
	Completeness check	5	All items except those required for adaptive questioning were mandatory. Respondents were required to complete the mandatory item before proceeding to the next page.
	Review step	5	Respondents were unable to change their responses once submitted.
**Response rates**	Unique site visitor	4	Each participant was allowed to take part in the survey only once by identifying the client computer's cookie and IP address.
	View rate (Ratio of unique survey visitors/unique site visitors)	N/A	N/A

	Participation rate (Ratio of unique visitors who agreed to participate/unique first survey page visitors)	Additional file 1	A total of 1,212 panel members visited the first page of the survey. Of these, 712 were rejected because they did not meet the inclusion criteria. Finally, 500 respondents agreed to participate in the survey, resulting in a participation rate of 41.25%.
	Completion rate (Ratio of users who finished the survey/users who agreed to participate)	Additional file 1	All respondents who agreed to participate finished the survey, showing a completion rate of 100.0%.
**Preventing multiple entries from the same individual**	Cookies used	4	To prevent duplication, a cookie was set as a unique identifier, and duplicate entries were avoided by preventing users when accessing with the same cookie. The cookie was valid for 7 days.
	IP check	4	To prevent duplication, the IP address of the client computer was set as a unique identifier, and duplicate entries were avoided by preventing users when accessing with the same IP address. There was no time limit to block access from the same IP address.
	Log file analysis	N/A	Not used.
	Registration	N/A	Not used.
**Analysis**	Handling of incomplete questionnaires	7	Only completed questionnaires were included in the final dataset.
	Questionnaires submitted with an atypical timestamp	N/A	Not used
	Statistical correction	N/A	Not used.

*Reference: Eysenbach G. Improving the quality of Web surveys: the Checklist for Reporting Results of Internet E-Surveys (CHERRIES). *Journal of medical Internet research.* 2004;6(3):e34.
