# Supplementary material for: Women’s attitudes toward certification logos, labels, and advertisements for organic disposable sanitary pads: results from a multi-city cross-sectional survey
Source: BMC Womens Health. 2022 Jun 17;22:237. doi: 10.1186/s12905-022-01723-z (PMC9206355; doi:10.1186/s12905-022-01723-z)
Supplement: Supplementary file 3 — Additional file 3. Contains all the questions of the survey asked for the study participants. A file named "Survey questionnaire" [file 12905_2022_1723_MOESM3_ESM.pdf]

Supplement 3. Survey questionnaire

Socio-demographics

1. Age (years)

1) 20–29
2) 30–39
3) 40–49
4) 50–59
5) ≥60

2. Education level completed

1) High school or below
2) Junior college
3) University
4) Graduate school
5) Other

3. Residential area

1) Seoul
2) Incheon, Gyeonggi
3) Daejeon, Sejong, Chungnam, Chungbuk
4) Gwangju, Jeonnam, Jeonbuk
5) Busan, Ulsan, Gyeongnam
6) Daegu, Gyeongbuk
7) Gangwon
8) Jeju

Purchase behavior of OSPs

4. How did you first get to know about OSPs? (Multiple responses available)

1) Manufacturers' advertisements
1) Label statements described on the product packaging
2) Celebrity advertisements sponsored by manufacturers
3) Recommendations from others (family or buyers)
4) Other

5. How do you usually buy OSPs? (Multiple responses available)

1) In-store shopping
2) e-commerce market
3) Other

6. Of each 10 times that you buy disposable sanitary pads, how often do you choose OSPs?

(/ 10 times)

7. When you buy a disposable sanitary pad, what criteria do you use to determine that the product is organic? (Multiple responses available)

1) Product description statement on the product packaging
2) Certification logo on the product packaging
3) Ingredients described in the label on the product packaging
4) Promotional statement of the product advertisement
5) Other

8. When you buy an OSP, do you usually check the part that contains organic ingredients?

1) Yes
2) No

9. When you buy an OSP, what do you expect most?

1) Excellent absorbency
2) Comfortable to wear
3) Safety for the human body
4) Environmental friendliness
5) Other

Attitudes toward certification logos, labels, and advertisements

10. Do you know the meaning of the certification logos of OSP products?

1) Fully not aware
2) Not aware
3) Neither aware nor not aware
4) Aware
5) Fully aware

11. Do you trust certification logos on OSP products?

1) Very unlikely
2) Unlikely
3) Neutral (Skip question 11-1)
4) Likely (Skip question 11-1)
5) Very likely (Skip question 11-1)

11-1. Why do you mistrust certification logos on OSP products?

1) Do not aware of the standard used for each certification
2) Hard to know certification authorities
3) Do not aware of how government manages the certifications
4) Cannot find any differences from other products
5) Other

12. Do the certification logos of OSP products create a positive image for the product?

1) Very unlikely (Skip question 12-1)
2) Unlikely (Skip question 12-1)
3) Neutral
4) Likely
5) Very likely

12-1. In what aspects do the certification logos of OSP products create a positive image for the product?

1) Excellent absorbency
2) Comfortable to wear
3) Safety for the human body
4) Environmental friendliness
5) Other

13. Do the certification logos of OSP products make you to build trust in the product?

1) Very unlikely (Skip question 13-1)
2) Unlikely (Skip question 13-1)
3) Neutral
4) Likely
5) Very likely

13-1. In what aspects do the certification logos of OSP products make you to build trust in the product?

1) Excellent absorbency
2) Comfortable to wear
3) Safety for the human body
4) Environmental friendliness
5) Other

14. Do the certification logos of OSP products affect your decision to purchase the product?

1) Very unlikely
2) Unlikely
3) Neutral
4) Likely
5) Very likely

15. Do you read what the label says?

	Very unlikely	Unlikely	Neutral	Likely	Very likely
15-1. Expiration date					

15-2. Manufacturer/Distributor					
15-3. Ingredients					
15-4. Storage methods					
15-5. Cautious for use					

16. Do you understand what the label says?

	Very unlikely	Unlikely	Neutral	Likely	Very likely
16-1. Expiration date					
16-2. Manufacturer/Distributor					
16-3. Ingredients					
16-4. Storage methods					
16-5. Cautious for use					

17. Do you perceive the label's content to be important?

	Very unlikely	Unlikely	Neutral	Likely	Very likely
17-1. Expiration date					
17-2. Manufacturer/Distributor					
17-3. Ingredients					
17-4. Storage methods					
17-5. Cautious for use					

18. Do you trust labels of OSP products?

1) Very unlikely
2) Unlikely
3) Neutral (Skip question 18-1)

4) Likely (Skip question 18-1)
5) Very likely (Skip question 18-1)

19. Why do you mistrust labels of OSP product?

1) Difficult to understand the content of the label
2) Hard to trust the manufacturers and distributors
3) Distrust in government management and regulation on label
4) Cannot find any differences from other products
5) Other

20. Do the labels of OSP products create a positive image for the product?

1) Very unlikely (Skip question 20-1)
2) Unlikely (Skip question 20-1)
3) Neutral
4) Likely
5) Very likely

20-1. In what aspects do the labels of OSP products create a positive image for the product?

1) Excellent absorbency
2) Comfortable to wear
3) Safety for the human body
4) Environmental friendliness
5) Other

21. Do the labels of OSP products affect your decision to purchase the product?

1) Very unlikely
2) Unlikely
3) Neutral
4) Likely
5) Very likely

22. Have you ever seen or heard about advertisement for OSPs?

1) Yes
2) No (go to question 30)

23. How do you usually see or hear advertisements for OSPs?

1) Broadcast advertisements
2) Print advertisements
3) Internet advertisements
4) Celebrity advertisements
5) Other

24. What interests you the most when you see or hear advertisements for OSP products?

1) Manufacturers and distributors
2) Product functionality
3) Safety for the human body
4) Environmental friendliness
5) Other

25. Do you trust advertisements of OSP product?

1) Very unlikely
2) Unlikely
3) Neutral (Skip question 25-1)
4) Likely (Skip question 25-1)
5) Very likely (Skip question 25-1)

25-1. Why do you mistrust advertisements of OSP product?

1) Difficult to determine the proof for safety for the human body
2) Distrust in government management and regulation on advertisement
3) Difficult to trust evidence for the advertisement
4) Cannot find any differences from other products
5) Other

26. Do the advertisements of OSP products create a positive image for the product?

1) Very unlikely (Skip question 26-1)
2) Unlikely (Skip question 26-1)
3) Neutral
4) Likely
5) Very likely

26-1. In what aspects do the advertisements of OSP products create a positive image for the product?

1) Excellent absorbency
2) Comfortable to wear
3) Safety for the human body
4) Environmental friendliness
5) Other

27. Do the advertisements of OSP products make you to build trust in the product?

1) Very unlikely (Skip question 27-1)
2) Unlikely (Skip question 27-1)
3) Neutral
4) Likely
5) Very likely

28. In what aspects do the advertisements of OSP products make you to build trust in the product?

1) Excellent absorbency
2) Comfortable to wear
3) Safety for the human body
4) Environmental friendliness
5) Other

29. Do the advertisements of OSP products affect your decision to purchase the product?

1) Very unlikely
2) Unlikely
3) Neutral
4) Likely
5) Very likely

Demands on government and OSP companies

30. What should a company do to manage labeling and advertising of OSPs?

1) Clearly state the parts (e.g., cover, absorbent layer, wings, etc.) made from organic ingredients of OSPs in labeling/advertising
2) Clearly indicate on the packaging of the product so that consumers can easily recognize the harmful ingredients used in OSPs
3) Clearly provide ingredient information on the website so that consumers can check the harmfulness of the ingredient
4) Clearly provide evidence for obtaining the organic certification logos on the website
5) Other

31. What should government do to manage labeling and advertising of OSPs?

1) Strengthen company punishment for unfair organic labeling and advertising
2) Strengthen company education to prevent unfair labeling and advertising
3) Strengthen consumer education to make wise choices and consumption
4) Strengthen management regulations for the use of certification logos
5) Other
